# Real-World Adherence to Toxicity Management Guidelines for Immune-Related Adverse Events

**DOI:** 10.3390/curroncol29050252

**Published:** 2022-04-28

**Authors:** Arezou Teimouri, Laura V. Minard, Samantha N. Scott, Amanda Daniels, Stephanie Snow

**Affiliations:** 1 Department of Pharmacy, Nova Scotia Health, QEII Health Sciences Centre, Halifax, NS B3H 3A7, Canada; laurav.minard@nshealth.ca (L.V.M.); samanthan.scott@nshealth.ca (S.N.S.); amanda.daniels@nshealth.ca (A.D.); 2 QEII Health Sciences Centre, Division of Medical Oncology, Dalhousie University, Halifax, NS B3H 4R2, Canada; stephanie.snow@nshealth.ca

**Keywords:** immune-related adverse events, immune checkpoint inhibitors, quality assurance, toxicity guideline adherence

## Abstract

Immune checkpoint inhibitors (ICIs) affect immunologic homeostasis, leading to immune-related adverse events (irAEs). Early irAE detection and management can prevent significant morbidity and mortality. A retrospective chart review was performed to characterize irAEs associated with nivolumab, ipilimumab, and nivolumab plus ipilimumab in adult medical oncology patients in Nova Scotia Health-Central Zone from 2013–2020, and to describe adherence to toxicity management guidelines. Diarrhea/colitis, hepatitis, pneumonitis, nephrotoxicity, and cardiotoxicity were studied. Of 129 charts reviewed, 67 patients (51.9%) experienced at least one irAE for a total of 98 irAEs and a 1.5% fatality rate. Of these irAEs, 33.7% led to an emergency room visit. Patients were admitted to hospital and steroids were used in 24.5% and 35.7% of cases, respectively. In 17.3% of irAEs, ICIs were permanently discontinued. In 20.4% of irAEs, ICIs were held, and patients were monitored; while in 18.4%, ICIs were held until the irAE was Grade 0–1 (and until steroids were tapered). Almost 47% of irAEs were managed according to guidelines (14.3% were not), and 38.8% had no documented management. Patients receiving immunotherapy frequently experience irAEs with half of irAEs having documented management adhering to guidelines. As immunotherapy indications expand, it is important to ensure irAEs are documented and managed appropriately.

## 1. Introduction

In the past several years, the field of oncology has evolved rapidly. While traditional cytotoxic chemotherapy and targeted medications continue to be used, treatment options for many cancers now include immunotherapy agents, or immune checkpoint inhibitors (ICIs). By blocking checkpoint proteins, ICIs allow the immune system cells (T cells) to better identify and destroy cancerous cells [1]. Drugs in this class bind to the checkpoint proteins/receptors CTLA-4 (ipilimumab, tremelimumab), PD-1 (pembrolizumab, nivolumab, cemiplimab), or PD-L1 (atezolizumab, durvalumab, avelumab) [1]. Due to their mechanism of action, ICIs can affect immunologic homeostasis and thus lead to immune-related adverse events (irAEs) [1]. Immune-related adverse events can affect any healthy tissue, causing inflammation and organ dysfunction [1,2,3]. For example, toxicities may include dermatological, hepatic, renal, neurotoxicity, cardiotoxicity, diarrhea and colitis, endocrinopathies, pneumonitis, and inflammatory arthritis [1].

Systematic reviews (SRs) and meta-analyses of randomized controlled trials (RCTs) have shown an increased risk of irAEs with CTLA-4, PD-1, and PD-L1 inhibitors versus non-immunotherapy control treatments [2,3,4]. Hypothyroidism, colitis, and pneumonitis were some of the most common irAEs identified in randomized trials [2,3,4]. A SR of prospective clinical trials similarly showed that endocrine (thyroid disorders), gastrointestinal (diarrhea, colitis, nausea), lung (pneumonitis), and musculoskeletal were the most frequently reported sites of irAEs [5]. Meta-analyses have also shown that anti-PD-1 agents (e.g., nivolumab) in combination with anti-CTLA-4 agents (e.g., ipilimumab) have a higher risk for adverse events [6,7,8,9,10,11].

In addition to information about irAEs from clinical trials, there has been interest in characterizing incidence and severity in real-world populations [12,13,14]. A large retrospective review of the Vigilyze-Vigibase (the World Health Organization database of adverse drug reactions) showed 613 fatal irAEs out of 31,059 ICI-related case reports (2.0%) [13]. The most frequent cause of fatal events with CTLA-4 inhibitors was colitis, while pneumonitis, hepatitis, and neurotoxic adverse effects were most likely associated with fatal events with PD-1/PD-L1 inhibitors [13]. Fatality rates were highest with myocarditis (39.7%) while only 2–5% for colitis and endocrine irAEs [13].

Early detection and management of irAEs are necessary to prevent significant morbidity and mortality [15]. Commonly, irAEs occur early during treatment, often within weeks to three months of receiving the ICI [16]. However, there have been reports of irAEs occurring up to one year after therapy discontinuation [16]. Multiple guidelines have been developed that outline how to screen for, diagnose, and manage these toxicities [1,15,16,17,18]. Grading of irAEs is typically based on the National Cancer Institute’s Common Terminology Criteria for Adverse Events (CTCAE) classification, with the severity of irAEs divided into grades 1–5 [1,16,19]. Guidelines, however, may still differ in their definitions of classifications, such as the case with grade 1 nephritis [1,15,16,17]. Despite an overall lack of evidence for how to best manage toxicities, guidelines [1,15,16,17,18] aim to aid clinicians in the detection and management of irAEs. There is, however, no local or national consensus regarding which set of guidelines should be used.

With mild irAEs, treatment with ICIs can usually continue, and increased monitoring may be recommended [15]. However, early detection is critical as moderate to severe irAEs can lead to significant morbidity and may be fatal [15]. Generally, with severe irAEs (grades 3–4), the ICI is discontinued [15,16]. Guidelines also recommend discontinuation of ICIs with some grade 2 irAEs [15]. Depending on the severity of the irAE, management may include the initiation of immunosuppressants such as high-dose corticosteroids, or immunomodulating drugs such as tumor necrosis factor-alpha (TNFa) antagonists [16].

While irAEs are well-characterized in clinical trials and with observational data, there is insufficient literature on whether real-world management of these irAEs adheres to clinical practice guidelines. A retrospective observational study of patients with a confirmed diagnosis of stage III/IV melanoma, treated with ipilimumab 3 mg/kg every three weeks, aimed to report on the management and outcomes of the irAE colitis (diarrhea) [20]. Although this study reviewed the recommendations of practice guidelines and discussed whether the management of irAEs in the cohort was in line with these recommendations, this was not an outcome of the study. Additionally, this study focused specifically on colitis with ipilimumab [20]. It is important to review and analyze the management of other irAEs in accordance with guideline recommendations to identify ways to improve patient care.

In this study, it was hypothesized that the increasing use of ICIs in oncology patients in Nova Scotia Health-Central Zone (NSH-CZ) has led to irAEs that require proper characterization, early detection, and appropriate management. By characterizing these toxicities and their management in a real-world setting, opportunities for quality improvement and to enhance patient care can be identified. As studies have shown a higher risk of adverse events with anti-PD-1 agents (e.g., nivolumab) in combination with anti-CTLA-4 agents (e.g., ipilimumab) [6,7,8,9,10,11], this study has categorized toxicities with the nivolumab and ipilimumab combination, as well as with each agent alone. This study categorized irAEs (diarrhea/colitis, hepatitis, pneumonitis, nephrotoxicity, and cardiotoxicity) associated with nivolumab, ipilimumab, or nivolumab plus ipilimumab and compared the management of irAEs to current ICI toxicity management guidelines.

## 2. Materials and Methods

### 2.1. Study Design

A retrospective chart review of the medical oncology pharmacy department and electronic records, at a single center, the Victoria General Hospital, was performed to characterize irAEs (by grade, by organ, by likelihood) associated with nivolumab, ipilimumab, and the nivolumab plus ipilimumab combination. The irAEs reviewed were diarrhea/colitis, hepatitis, pneumonitis, nephrotoxicity, and cardiotoxicity. Due to the difficulty in ascertaining the exact cause of an adverse event in some cases (e.g., radiation pneumonitis versus immune-mediated pneumonitis), irAEs were categorized as ‘definitely’, ‘probably’, or ‘possibly’ based on clinical evidence and/or physician documentation. The chart review analyzed how these irAEs were managed and whether this management adhered to current guidelines [1,15,16,17,18]. Grading and management of irAEs for data collection was based on a compilation of the Cancer Care Ontario, American Society of Clinical Oncology (ASCO), European Society of Medical Oncology (ESMO), National Comprehensive Cancer Network (NCCN), and British Columbia (BC) Cancer guidelines (Table A1) [1,15,16,17,18]. Grading of irAEs (Table A2) was primarily based on the Cancer Care Ontario guidelines [1]. Cardiovascular irAEs were based on the NCCN guidelines [17] as cardiovascular toxicities were not included in as much detail in all guidelines. The management of an irAE was considered to have adhered to guidelines if it followed at least one of these five guidelines. Additionally, as these guidelines were published after our study period start date of 2010, guideline adherence prior to and after the publication of included guidelines (2017) was also reviewed. Ethics approval was obtained from the Nova Scotia Health Research Ethics Board (REB# 1026670). The requirement for informed consent was waived by the board.

### 2.2. Participants

Participants were identified using two lists that are maintained by the pharmacy department: (1) the Department of Health and Wellness New Cancer Drug Fund approvals list and (2) the list of patients who have received an ICI as part of a pharmaceutical company-sponsored patient support program (PSP). Adult medical oncology patients (≥18 years of age) with an NSH-CZ postal code who had received at least one dose of nivolumab, ipilimumab, or the nivolumab plus ipilimumab combination for cancer treatment at the Victoria General Hospital were selected for this study. Patients must have received the ICI through a PSP or with funding from the Department of Health and Wellness, between 1 January 2010 and 31 December 2020. Patients who switched from another ICI to these agents were also included in the study (*n* = 1). Patients receiving immunotherapy agents in combination with chemotherapy or targeted therapy were excluded due to the potential for overlap in toxicity profiles that could confound results. Patients receiving immunotherapy as part of a clinical trial or as compassionate supply outside of a PSP were also excluded.

### 2.3. Outcomes

The primary outcomes were to (a) categorize irAEs (diarrhea/colitis, hepatitis, pneumonitis, nephrotoxicity, and cardiotoxicity) associated with nivolumab, ipilimumab, or nivolumab plus ipilimumab by organ, grade and likelihood and (b) compare the management of irAEs to current ICI toxicity management guidelines.

### 2.4. Data Collection and Analysis

Medical and electronic records were utilized to characterize irAEs. Data collected included sex, age, and time to and cause of death (if applicable). Immunotherapy-related data collection included ICI drug name, dose, cycle frequency, number of cycles of treatment, indication (cancer diagnosis), emergency room visit, and/or hospital admission for irAE (if applicable), and time to and cause of ICI discontinuation (where applicable). Time to first documentation of irAE (from the first cycle) was also collected. Grading and management of irAEs for data collection were based on a compilation of available guidelines [1,15,16,17,18] (Table A2). Grading of irAEs (Table A2) was primarily based on the Cancer Care Ontario guidelines [1]. Cardiovascular irAEs were based on the NCCN guidelines [17].

Descriptive statistics were utilized for the characterization of irAEs. Immune-related adverse events were categorized into ‘definitely’, ‘probably’, or ‘possibly’. The number of irAEs was aggregated by organ/system and by severity grade. Frequency and percentages were used for categorical variables (e.g., sex). Means and standard deviations were utilized for continuous variables (e.g., age). The median and interquartile ranges were used for the number of cycles, time to death, time to discontinuation, and time to first documentation of irAE (from the first dose of ICI). Analysis of toxicity management and guideline adherence was descriptive. Management of ‘clinically relevant’ irAEs (≥grade 2) was also reviewed. Fatality rates were calculated as the number of fatal events divided by the total number of patients who received ICIs. Only fatal events that were thought to be due to the irAEs of interest were included in the fatality rate. Microsoft Excel was utilized for data collection and statistical analyses.

## 3. Results

### 3.1. Patient Characteristics

**Figure 1:**
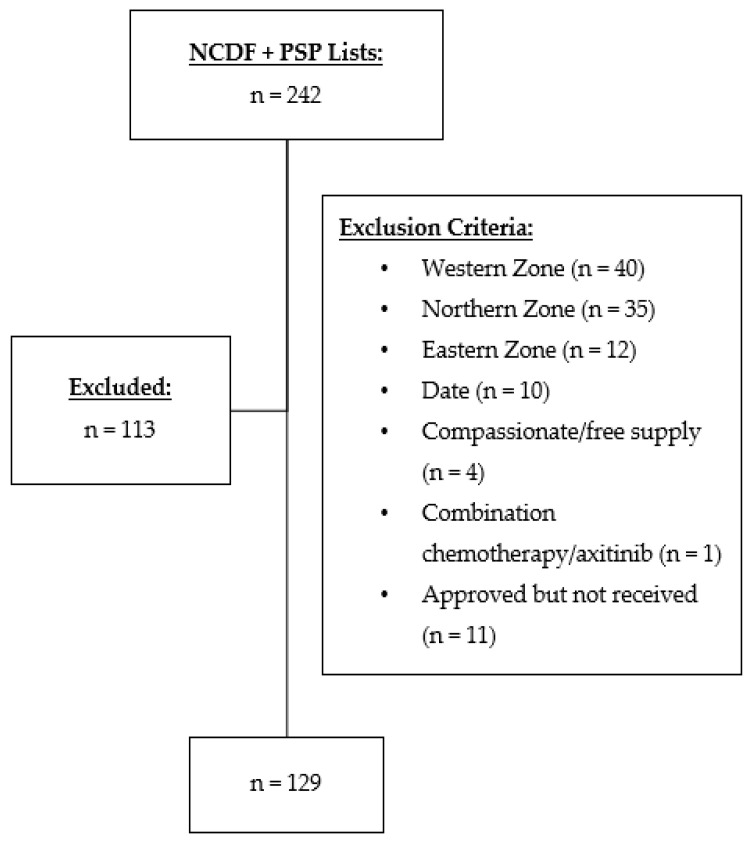
Participant selection flow diagram of medical oncology patients receiving nivolumab, ipilimumab, or nivolumab plus ipilimumab at Nova Scotia Health-Central Zone between 2010–2020. NCDF: New Cancer Drug Fund; PSP: Patient Support Program.

A total of 242 patients were identified who received nivolumab, ipilimumab, or nivolumab plus ipilimumab for cancer treatment (Figure 1). Of those, 113 were excluded, with 129 total charts included in this retrospective review (Figure 1). Baseline characteristics are presented in Table 1. The average age of patients was 64 (±11) with more males included in the study than females. The most common indication for ICI use was metastatic non-small cell lung cancer. All patients had advanced disease, and none were being treated with curative intent.

### 3.2. Characterization of Immune-Related Adverse Events

A total of 51.9% (67/129) of patients experienced at least one new irAE for a total of 98 irAEs. Of the 98 irAEs, 25.5% were ‘definitely’, 8.2% ‘probably’, and 66.3% ‘possibly’ caused by the ICI(s). There were 42 new irAEs in the nivolumab plus ipilimumab group, 48 in the nivolumab group, and 8 in the ipilimumab group (Table 2). There were more events rated as ‘definitely’ caused by an irAE in the combination group versus the ipilimumab or nivolumab groups (Table 2). The most common irAE was diarrhea/colitis, followed by hepatitis, pneumonitis, nephrotoxicity, and cardiotoxicity (Table 3). Most diarrhea/colitis (25/41), hepatitis (18/28), and nephrotoxicity (7/12) irAEs were grade 1, while most pneumonitis (8/13) was grade 2 and most cardiotoxicities (3/4) were grade 4 (Table 3).

**Figure 2:**
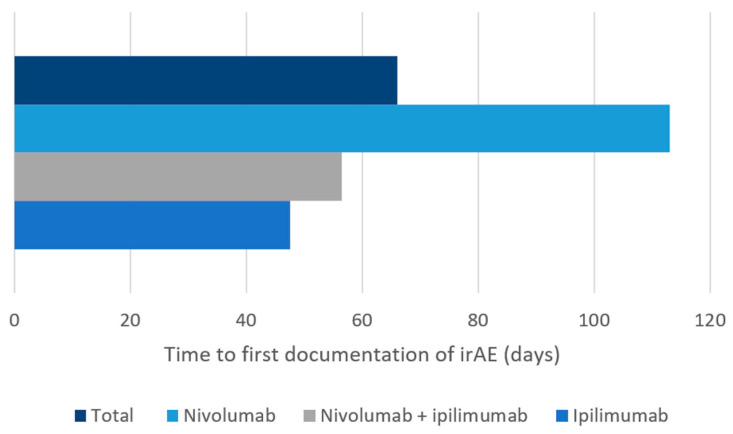
Median time to first immune-related adverse event documentation from the first dose of nivolumab, ipilimumab, or nivolumab plus ipilimumab, in medical oncology patients at Nova Scotia Health-Central Zone. irAE: immune-related adverse event.

Overall, 33.7% (33/98) of the new irAEs led to an ER visit. There were 52.4% (22/42) ER visits in the nivolumab plus ipilimumab group, 20.8% (10/48) in the nivolumab group, and 12.5% (1/8) in the ipilimumab group (Table 2). In patients who received nivolumab plus ipilimumab, most diarrhea/colitis events were grade 2, and almost two-thirds of patients presented to the ER (Table 3). Most diarrhea/colitis in the single-agent nivolumab and ipilimumab groups was grade 1, however, with only one ER visit in the nivolumab group (Table 3). In all groups, most hepatitis events were grade 1 (47–89%), although there were more ER visits (33.3%) in the nivolumab plus ipilimumab group (Table 3). Pneumonitis was most commonly reported in patients receiving nivolumab with the majority being grade 2 and two-thirds resulting in an ER visit (Table 3). Most nephrotoxicity irAEs were reported in patients receiving nivolumab with all but one event being grade 1; however, nephrotoxicity events in the nivolumab plus ipilimumab group were most likely to be grade 2 with three out of four patients requiring an ER visit. All four patients with immune-related cardiotoxicity reported in this study (two in the nivolumab plus ipilimumab group and two in the nivolumab group) presented to the ER and three of these were grade 4. There was no pneumonitis or cardiotoxicity reported with single-agent ipilimumab. The median time to first irAE documentation (from the first dose of ICI) ranged from 48 to 113 days (Figure 2).

Of 129 patients, 68 (52.7%) discontinued therapy and 57 (44.2%) died during the study period (Table 4). Drug inefficacy and/or disease progression was the most common cause of discontinuation of therapy (55.9%) or death (89.5%). Sixteen patients discontinued therapy and two died (fatality rate 1.5%) due to an irAE of interest. Pneumonitis and colitis were common causes of discontinuation related to irAEs. Both cases of death due to an irAE of interest were in the nivolumab plus ipilimumab group and were considered to be ‘definitely’ caused by an irAE. Both cases were renal cell carcinoma and irAEs were managed according to guidelines. The cause of death was colitis in one case after three cycles of therapy: time to toxicity was 38 days and time to death was 200 days. Hepatitis, nephrotoxicity, and cardiotoxicity were causes of death in the other case with two cycles of therapy: time to toxicity and time to death were 23 and 33 days, respectively. The median time to death and to discontinuation for any reason was 155 (IQR 261) and 109 (IQR 209) days, respectively.

### 3.3. Management of Immune-Related Adverse Events

**Figure 3:**
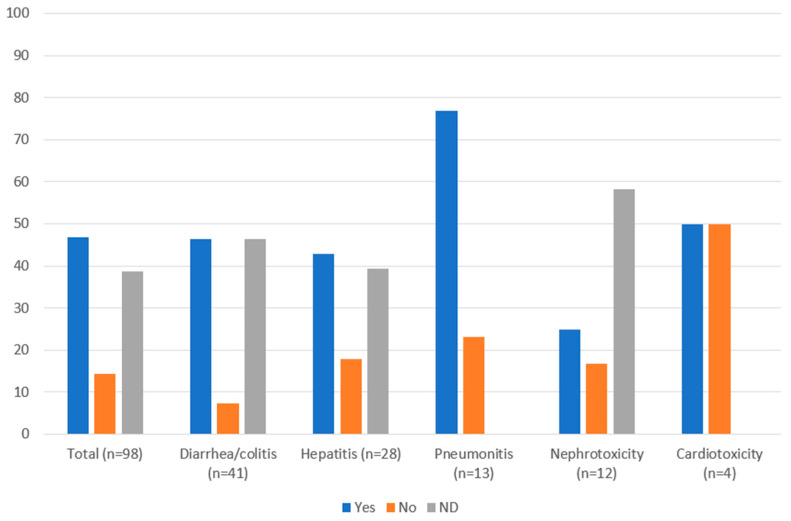
Guideline adherence for management of immune-related adverse events (%) in patients with cancer receiving nivolumab, ipilimumab, or nivolumab plus ipilimumab in Nova Scotia Health-Central Zone, by organ system. ND: not documented.

Management of irAEs was in accordance with guidelines in 46.9% of cases, not in accordance with guidelines in 14.3% of cases and 38.8% had no documented management (Figure 3). Many of the cases where management was not documented were low grade and may have been appropriately managed through observation. Management of clinically relevant irAEs (≥grade 2) was more likely to adhere to guidelines: 68.9% (31/45) were managed in accordance with guidelines, 22.2% (10/45) were not, and 8.9% (4/45) had no documented management. Management of all irAEs was also analyzed by organ system (Figure 3). Diarrhea/colitis and hepatitis were managed in accordance with guidelines in 46.3% and 42.9% of cases, respectively. Most pneumonitis cases (76.9%) were managed according to guidelines, while for nephrotoxicity, only 25.0% of cases were managed in accordance with guidelines. Cardiotoxicity events were managed according to guidelines in half of the cases. There was no documented management in 46.3%, 39.3%, and 58.3% of diarrhea/colitis, hepatitis, and nephrotoxicity cases, respectively.

When reviewing irAEs managed prior to and after guideline publication (2017), it was identified that 10 of 98 irAEs were documented from 2013–2016. Of these 10 irAEs, seven were managed according to guidelines, one was not, and two had no documented management. For irAEs documented from 2017 onwards, 44.3% (39/88) were managed according to guidelines, 14.8% (13/88) were not, and 40.9% (36/88) had no documented management.

Table 5 shows the various ways in which irAEs were managed by organ system, including 24 irAEs that led to hospital admissions. Steroids were used to manage irAEs in 35 of 98 cases. Immune checkpoint inhibitors were held, and patients were monitored in 20 cases, while ICIs were held until the irAE was Grade 0–1 (and until steroids were tapered) in 18 cases, and ICIs were permanently discontinued in 17 cases. When reviewing the 17 irAEs where ICIs were permanently discontinued, five were due to colitis (one patient died), three due to hepatitis, six due to pneumonitis, two due to nephrotoxicity, and one due to cardiotoxicity. One of these patients died due to hepatitis, pneumonitis, and nephrotoxicity.

Of the 45 clinically relevant irAEs (≥grade 2), 10 (22.2%) were not managed according to guidelines. In seven of these 10 cases, therapy was continued despite guideline suggestions to hold or discontinue therapy. In two cases, therapy was held but steroids were not started, and/or regular monitoring was not completed as per guidelines. In one case, management at grade 2 was not initiated and the irAE progressed to grade 3. Of these 10 irAEs, seven were grade 2, one was grade 3, and two were characterized as grade 4. Two of these patients died; however, in both cases, the cause of death was documented as disease progression.

## 4. Discussion

Immune checkpoint inhibitor use is increasing throughout cancer care. Therefore, there is a greater need to better understand and manage irAEs with ICIs in the real world due to the high risk of morbidity and mortality with these events. Literature has shown that ICIs used in combination, such as nivolumab plus ipilimumab, increase the risk for adverse events [6,7,8,9,10,11]. Consistent with this, the current study showed more ‘definitely’ irAEs and ER visits in the nivolumab plus ipilimumab group (Table 2). Diarrhea/colitis was the most common irAE identified in this study, which is consistent with other publications [3]. The fatality rate reported here (1.5%) was in line with other reported fatality rates of 2.0% (613 fatal irAEs/31,059 ICI-related case reports) [13] and 0.64% (42 fatal irAEs/6528 patients receiving ICIs) [3]. Both fatalities reported were in patients who had received nivolumab plus ipilimumab: one due to hepatitis/nephritis/cardiotoxicity and one due to colitis. One SR found that the most common cause of fatal irAEs was colitis with ipilimumab [3]. In a large retrospective review, the most common cause of fatal irAEs was myocarditis and colitis with anti-CTLA-4 agents and pneumonitis with anti-PD-1/PD-L1 agents [13].

To our knowledge, this is the first study to include guideline adherence as an outcome and to report the proportion of irAEs that were managed according to guidelines. This study provides insight into real-world irAE management guideline adherence, with 14.3% of all irAEs and 22.2% of clinically relevant irAEs not managed according to guidelines. Of these clinically relevant irAEs, two patients died during the study period; however, these deaths were documented as due to disease progression. Almost 40% of all irAEs and 8.9% of clinically relevant irAEs (≥grade 2) had no documented management. Reasons for a lack of documentation may be related to the grade of irAEs: most were grade 1 and may have been monitored/managed without official documentation as part of the patient record. In support of this idea, when only clinically relevant irAEs were reviewed, there were fewer cases where documentation was lacking.

Despite an overall lack of evidence for how to best manage toxicities, guidelines [1,15,16,17,18] aim to aid clinicians in the detection and management of irAEs. There is, however, no local or national consensus regarding which set of guidelines should be used. Guidelines for irAEs are rapidly evolving and becoming more site-specific with the involvement of expert subspecialists. Most oncology guidelines, including those utilized in this study, are derived from clinical trial protocols and are not necessarily evidence-based. The cornerstone of management of non-endocrine irAEs that are severe enough to require intervention has been with systemic corticosteroids dosed at 1–2 mg/kg of prednisone or intravenous (IV) equivalent. In this study, 35 of 98 irAEs (35.7%) were treated with steroids. A retrospective observational study of patients treated with ipilimumab 3 mg/kg every three weeks, showed that half of the patients with colitis were managed with high-dose prednisone [20]. This study with ipilimumab, nivolumab, and nivolumab plus ipilimumab, showed that colitis was treated with steroids in 13 of 41 cases (31.7%). Of the five irAEs studied, the irAE most frequently treated with steroids was pneumonitis (69.2%; 9/13 cases), which was grade 2 or 3 in most identified cases. While steroids are generally recommended for grade ≥ 2 irAEs, this recommendation may change with increasing evidence on individualized management of irAEs. The potential adverse effects of high-dose steroids have been well known for decades [21]. Thus, there is a greater need for guidelines for oncologists on how to manage the adverse effects of the irAE management itself (e.g., bone protection, pneumocystis pneumonia infection prevention, gut protection).

Further, the data about the potential impact of corticosteroids on the efficacy of ICI therapy is mixed, and some data suggest that patients who are receiving corticosteroids as management for irAEs may have worse outcomes from a cancer perspective [22]. This may result in the reluctance of some physicians to use high-dose corticosteroids, even if that is the recommendation made in a treatment guideline. Specifically, a 2018 retrospective study reported that the use of low-dose versus high-dose steroids for irAEs was associated with prolonged time to treatment failure and overall survival [23]. Additionally, in the KEYNOTE 054 clinical trial, the treatment effect was noted to be lower in the ICI arm after systemic steroid use for irAEs versus no steroid use (hazard ratio 0.5 vs. 0.34) [22].

With time and experience, we have come to understand that the immunomodulatory cause underlying different irAEs may vary. For instance, some irAEs, including myocarditis and toxic epidermal necrolysis, have been recognized as arising when T-cells react to antigens in healthy tissue, while other irAEs including colitis and arthritis develop via cytokine-mediated mechanisms. Other irAEs occur in the setting of antibody-mediated inflammation, including several immune-mediated neurologic conditions and bullous pemphigoid [24,25]. Indeed, this has led to a call for integration of this knowledge to develop a personalized decision-making process in the management of each patient, moving away from the monolith approach of 1 mg/kg of prednisone for all in some cases.

Progress has been made in this direction in large part with the engagement of subspecialists who are experts in the body system in which the irAE arises. For example, several irAE management guidelines in subspecialty literature outside oncology have already been published: gastroenterology [26], dermatology [27], endocrinology [28], and rheumatology [29]. Many of the interventions outlined in these guidelines stem from that subspeciality’s expertise in the management of analogous autoimmune conditions that irAEs mimic, with the recommendation for use of many steroid-sparing DMARDs which are largely not medications that the average oncologist has experience with administering. Thus, ongoing collaboration with subspecialists and referral of patients with irAEs severe enough to necessitate intervention is strongly encouraged to foster interdisciplinary research and provide best patient care. For instance, Chen et al. report that dermatology consultation reduces interruption of oncologic management among hospitalized patients with irAEs [30].

As an observational, retrospective study, this study is limited in design and prone to biases associated with retrospective studies. Inconsistent documentation and the inability of the paper and electronic medical records to capture all data (e.g., patients who were monitored with lower grade irAEs) also limit the results. There was a focus on irAE characterization in patients with cancer receiving nivolumab plus ipilimumab, nivolumab or ipilimumab in NSH-CZ. The decision to only review these agents, and therefore, omitting all other ICIs and their indications, may have skewed results, in terms of both the patients being treated and individual differences in practice between different oncologists. Despite this, there were no other CTLA4 inhibitors in use in NSH-CZ outside of clinical trials, and data suggests similar rates of irAEs for all anti-PD-1/PD-L1 inhibitors. Additionally, different prescribers may manage irAEs differently, and these results may, therefore, not be reflective of irAE management by all oncology prescribers. Similarly, the decision to only review five of the most common irAEs may have limited findings. Patients were excluded if they received therapy in satellite chemotherapy units and analysis was limited to patients being treated and followed at NSH-CZ, which is a university-affiliated academic center where management would have been overseen presumably by a medical oncologist. Patients treated in the community have more care provided by general practitioners in oncology (GPOs) who have a different experience and skill set, and hence these results may be less generalizable outside of the academic center. Categorization of irAEs as ‘definitely’, ‘probably’, or ‘possibly’ was also difficult to ascertain at times (e.g., hepatic metastases versus immune-related hepatitis). Where possible, this was controlled by utilizing clinical evidence and team members’ clinical expertise to categorize irAEs. Furthermore, this study was not designed to complete statistical analyses or comparisons. Therefore, no statistical inferences can be made from the results. Finally, the earliest patients included were treated at the advent of ICI therapy, at the beginning of the learning curve for recognition and management of irAEs. Indeed, the guidelines used to gauge adherence to recommended irAE management in this study were all published later than the earliest patients were treated, and with each iteration of irAE management guidelines, recommendations are becoming more refined as more data is available to inform their content.

This study focused on irAE characterization in patients with cancer receiving nivolumab plus ipilimumab, nivolumab, or ipilimumab at a single center. The results of this study can be utilized to increase patient safety, improve patient outcomes, and increase patients’ quality of life through increasing awareness, and thus better detection and management of irAEs. In the next five years, ICI use is likely to increase, and therefore better detection and management of irAEs are required. Knowledge gaps with regards to clinical irAE management currently exist, with oncology guidelines based on clinical trial protocols and consensus. Researchers may consider data from future real-world studies for organ system-specific irAE management. Further research can expand to include other commonly used ICIs such as pembrolizumab as well as irAEs that affect other organ systems (e.g., endocrine toxicities, cutaneous toxicities) to provide further insight into the incidence of these irAEs and their management in NSH-CZ. Future interventions to improve irAE management and documentation may include system-specific standardized order sets outlining how to manage each irAE, as well as pharmacists working in multidisciplinary clinics and being involved in irAE management and documentation. It will also be important to analyze the impact of such interventions on patient care. Including data from patients treated outside of the academic center may also offer unique and interesting insights and increase the generalizability of the results. Additionally, more quality assurance research could be done prospectively to inform best practice for utilizing broad and site-specific guidelines.

## Figures and Tables

**Table 1:** Baseline characteristics of Nova Scotia Health-Central Zone medical oncology patients receiving nivolumab, ipilimumab, or nivolumab plus ipilimumab from 2013–2020 (*n* = 129).

Characteristic	*n* = 129
Age (mean ± SD)	64 ± 11
Sex [*n* (%)]	
Male	84 (65.1)
Female	45 (34.9)
Cancer Type [*n* (%)]	
Melanoma	32 (24.8)
Renal Cell Carcinoma	38 (29.5)
Non-Small Cell Lung Cancer	51 (39.5)
Squamous Cell Carcinoma of Head and Neck	8 (6.2)
Drug [*n* (%)]	
Nivolumab + Ipilimumab	41 (31.8)
Nivolumab	78 (60.5)
Ipilimumab	10 (7.7)
Dosing [*n* (%)]	
Nivolumab 1 mg/kg + Ipilimumab 3 mg/kg	17 (13.2)
Nivolumab 3 mg/kg + Ipilimumab 1 mg/kg	24 (18.6)
Nivolumab 3 mg/kg	19 (14.7)
Nivolumab 6 mg/kg	7 (5.4)
Nivolumab Fixed Dosing ^a^	52 (40.3)
Ipilimumab 3 mg/kg	10 (7.8)
Number of Cycles (median ± IQR) ^b^	
Nivolumab + Ipilimumab	4 ± 10
Nivolumab	4 ± 5.8
Ipilimumab	3.5 ± 1

IQR: interquartile range. ^a^ 480 mg every 28 days, exception: one patient received 240 mg every 28 days. ^b^ The number of cycles (range) was: nivolumab + ipilimumab (1–59); nivolumab (1–43); ipilimumab (1–4).

**Table 2:** Total immune-related adverse events in patients receiving nivolumab plus ipilimumab, nivolumab, or ipilimumab, categorized by likelihood and emergency room visits.

Drug	irAE Likelihood (*n* [%])			ER Visit (*n* [%])
	Definitely	Probably	Possibly	
Total (*n* = 98)	25 (25.5)	8 (8.2)	65 (66.3)	33 (33.7)
Nivolumab + Ipilimumab (*n* = 42)	20 (47.6)	6 (14.3)	16 (38.1)	22 (52.4)
Nivolumab (*n* = 48)	4 (8.3)	2 (4.2)	42 (87.5)	10 (20.8)
Ipilimumab (*n* = 8)	1 (12.5)	0	7 (87.5)	1 (12.5)

ER: emergency room; irAE: immune-related adverse event.

**Table 3:** New immune-related adverse events in Nova Scotia Health-Central Zone patients receiving nivolumab plus ipilimumab, nivolumab, or ipilimumab, categorized by organ system, toxicity grade, and emergency room visits.

Drug	Grade *n* (%)				ER Visit *n* (%)
	1	2	3	4	
Diarrhea/Colitis					
Total (*n* = 41)	25 (60.9)	10 (24.4)	5 (12.2)	1 (2.4)	12 (29.3)
Nivolumab + Ipilimumab (*n* = 17)	4 (23.5)	7 (41.2)	5 (29.4)	1 (5.9)	11 (64.7)
Nivolumab (*n* = 21)	18 (85.7)	3 (14.3)	0	0	1 (4.8)
Ipilimumab (*n* = 3)	3 (100)	0	0	0	0
Hepatitis					
Total (*n* = 28)	18 (64.3)	5 (17.8)	4 (14.3)	1 (3.6)	6 (21.4)
Nivolumab + Ipilimumab (*n* = 15)	7 (46.7)	3 (20.0)	4 (26.7)	1 (6.6)	5 (33.3)
Nivolumab (*n* = 9)	8 (88.9)	1 (11.1)	0	0	1 (11.1)
Ipilimumab (*n* = 4)	3 (75.0)	1 (25.0)	0	0	0
Pneumonitis ^a^					
Total (*n* = 13)	2 (15.4)	8 (61.5)	3 (23.1)	0	7 (53.8)
Nivolumab + Ipilimumab (*n* = 4)	1 (25.0)	2 (50.0)	1 (25.0)	0	1 (25.0)
Nivolumab (*n* = 9)	1 (11.1)	6 (66.7)	2 (22.2)	0	6 (66.6)
Nephrotoxicity					
Total (*n* = 12)	7 (58.3)	5 (41.7)	0	0	4 (33.3)
Nivolumab + Ipilimumab (*n* = 4)	1 (25.0)	3 (75.0)	0	0	3 (75.0)
Nivolumab (*n* = 7)	6 (85.7)	1 (14.3)	0	0	0
Ipilimumab (*n* = 1)	0	1 (100)	0	0	1 (100)
Cardiotoxicity ^a^					
Total (*n* = 4)	1 (25.0)	0	0	3 (75.0)	4 (100)
Nivolumab + Ipilimumab (*n* = 2)	1 (50.0)	0	0	1 (50.0)	2 (100)
Nivolumab (*n* = 2)	0	0	0	2 (100)	2 (100)

ER: emergency room, ^a^ There were no events in the ipilimumab group.

**Table 4:** The proportion of medical oncology patients in the Nova Scotia Health-Central Zone treated with nivolumab, ipilimumab, or nivolumab plus ipilimumab who discontinued treatment or died between 2013–2020.

	*n* = 129
Discontinuation [*n* (%)]	68 (52.7)
Inefficacy/Disease Progression	38 (55.9)
Toxicity (irAE of interest) ^a^	16 (23.5)
Pneumonitis	8 (50.0)
Colitis	7 (43.8)
Nephrotoxicity	1 (6.25)
Toxicity (other)	4 (5.9)
Other	10 (14.7)
Death [*n* (%)]	57 (44.2)
Disease Progression	51 (89.5)
Toxicity (irAE of interest) ^a^	2 (3.5)
Hepatitis, nephrotoxicity, cardiotoxicity	1 (50.0)
Colitis	1 (50.0)
Toxicity (other)	0
Other	4 (7.0)

^a^ diarrhea/colitis, hepatitis, pneumonitis, nephrotoxicity, cardiotoxicity.

**Table 5:** Aggregated data for the management of immune-related adverse events in Nova Scotia Health-Central Zone patients treated with nivolumab plus ipilimumab, nivolumab, or ipilimumab, categorized by organ system.

Management (*n* [%])	Diarrhea/Colitis (*n* = 41)	Hepatitis (*n* = 28)	Pneumonitis (*n* = 13)	Nephrotoxicity (*n* = 12)	Cardiotoxicity (*n* = 4)
Therapy continued and monitored	7 (17.1)	3 (10.7)	2 (15.4)	2 (16.7)	0
Steroids:	13 (31.7) ^a^	8 (28.6)	9 (69.2)	3 (25.0)	2 (50.0)
Prednisone 0.5–1 mg/kg/day ^b^	9 (22.0)	4 (14.3)	8 (61.5)	1 (8.3)	0
Prednisone 2 mg/kg/day ^b^	1 (2.4)	2 (7.1)	1 (7.7)	1 (8.3)	2 (50.0)
Methylprednisolone 1–2 (or 2–4) mg/kg/day ^c^	5 (12.2)	2 (7.1)	0	1 (8.3)	0
Loperamide	7 (17.1)	--	--	--	--
Oral/IV hydration	6 (14.6)	--	--	4 (33.3)	--
Admitted to hospital	9 (22.0)	3 (10.7)	5 (38.5)	4 (33.3)	3 (75)
Empiric antibiotic therapy	5 (12.2)	--	--	--	--
Infliximab 5 mg/kg IV	1 (2.4)	--	0	--	--
Mycophenolate mofetil	--	2 (7.1)	--	--	--
Therapy held and monitored	4 (9.8)	6 (21.4)	6 (46.2)	0	4 (100)
Therapy held until Grade 0–1 and prednisone < 7.5 mg/day (anti-CTLA-4) or <10 mg/day (anti-PD-1)	8 (19.5)	6 (21.4)	3 (23.1)	1 (8.3)	0
Permanently discontinued therapy ^d^	5 (12.2)	3 (10.7)	6 (46.1)	2 (16.7)	1 (25.0)

^a^ All doses of prednisone or switches to methylprednisolone used per irAE were documented. Thus, the number of different steroid regimens is greater than 13, ^b^ Orally (or IV equivalent) then taper, ^c^ IV then taper, ^d^ Discontinuations were counted per irAE, not per patient, --: not applicable/recommended for that irAE.

## Data Availability

The data presented in this study are available within this article. Data has been de-identified and aggregated. Individual level data is not available due to patient confidentiality.

## Appendix A

**Table A1:** Brief overview of the five included guidelines [1,15,16,17,18].

	CCO [1]	ASCO [15]	ESMO [16]	NCCN [17]	BC Cancer [18]
Publication Year	2020	2018	2017	2019	2019
Nationality	Canadian	American	European	American	Canadian
Guideline Type	Clinical Practice Guideline	Clinical Practice Guideline	Clinical Practice Guideline	Clinical Practice Guideline	Protocol Summary
Methodology	Multidisciplinary working group of oncology clinicians with immunotherapy experience. Based on best available evidence, current practice in Ontario, guidance from clinical experts, and working group consensus.	Multidisciplinary, multi-organizational panel of experts in medical oncology, dermatology, gastroenterology, rheumatology, pulmonology, endocrinology, urology, neurology, hematology, emergency medicine, nursing, trialist, and advocacy. A systematic review of the literature (focused on guidelines, systematic reviews and meta-analyses, randomized controlled trials, and case series published from 2000 through 2017) and an informal consensus process.	Developed in accordance with the ESMO standard operating procedures for Clinical Practice Guidelines development. The relevant literature has been selected by the expert authors. Levels of evidence and grades of recommendation have been applied. Statements without grading were considered justified standard clinical practice by the experts and the ESMO Faculty.	A statement of evidence and consensus of the authors regarding their views of currently accepted approaches to treatment.	A statement of concensus of BC Cancer professionals regarding their views of currently accepted approaches to treatment.
Diarrhea and Colitis	Management algorithm for diarrhea/colitis. Includes supportive therapy approaches.	Managament of colitis outlined in bullet point form. Diagnostic work-up and supportive therapy included.	Management algorithm for diarrhea/colitis. Other assessment and investigations included.	Management algorithm for diarrhea/colitis. Supportive therapy included.	Management algorithm for enterocolitis (diarrhea, abdominal pain, mucus, or blood in stools with or without fever, ileus, peritoneal signs).
Pneumonitisi	Management algorithm for pneumonitis. Includes supportive therapy approaches.	Managament of pneumonitis outlined in bullet point form. Diagnostic work-up and supportive therapy included.	Management algorithm for pneumonitis. Other assessment and investigations included.	Management algorithm for pneumonitis. Workup/supportive therapy included.	Management algorithm for pneumonitis (radiographic changes, new or worsening cough, chest pain, shortness of breath).
Hepatitis	Management algorithm for hepatitis. Includes supportive therapy approaches.	Managament for hepatitis outlined in bullet point form. Diagnostic work-up and supportive therapy included.	Management algorithm for hepatitis. Other assessment and investigations included.	Management algorithm for hepatic adverse events. Assessment/supportive therapy included.	Management algorithm for liver irAE (abnormal liver function test, jaundice, tiredness).
Nephrotoxicity	Management algorithm for renal toxicities. Includes supportive therapy approaches.	Management for nephritis and symptomatic nephritis outlined in bullet point form. Diagnostic work-up and supportive therapy included.	Management algorithm for nephritis. Other assessment and investigations included.	Management algorithm for renal adverse events (elevated serum creatinine/acute renal failure). Assessment/supportive therapy included.	Management algorithm for renal irAE (increase in serum creatinine, decreased urine output, hematuria, edema).
Cardiotoxicity	Brief review.	Management for cardiovascular toxicities outlined in bullet point form. Includes two sections: (1) myocarditis, pericarditis, arrhythmias, impaired ventricular function with heart failure and vasculitis, (2) venous thromboembolism. Diagnostic work-up and supportive therapy included.	Brief review.	Management algorithm for cardiovascular adverse events (myocarditis, pericarditis, arrhythmias, impaired ventricular function). Assessment/supportive therapy included.	Brief review of cardiovascular irAE (angiopathy, myositis, myocarditis, pericarditis, temporal arteritis, vasculitis).

**Table A2:** Immune-related adverse event grading definitions utilized for data collection [1,15,16,17,18].

	Diarrhea /Colitis	Pneumonitis	Hepatitis	Nephrotoxicity	Cardiotoxicity
Grade 1	<4 stools/day above baseline	Asymptomatic; diagnostic radiological observations only; no intervention needed	AST/ALT up to 3 × ULN or total bilirubin up to 1.5 × ULN (for <2 × baseline)	Creatinine > 1–1.5 × ULN or 1.5 × baseline	Asymptomatic, creatine kinase or troponin elevation, abnormal ECG or findings consistent with pericarditis
Grade 2	4–6 stools above baseline, abdominal pain, mucus or blood in stool	Symptomatic; medical intervention indicated, limiting instrumental ADL	AST/ALT > 3–5 × ULN or total bilirubin > 1.5–3 × ULN (or >2 × baseline)	Creatinine > 1.5–3.0 × ULN or >1.5–3.0 × baseline	Mild symptoms or symptoms with moderate activity/ exertion, abnormal screening tests
Grade 3	≥7 stools/day above baseline, need for hospitalization for IV fluids ≥ 24 h	Severe symptoms; limiting self-care ADL; oxygen indicated	AST/ALT > 5–20 × ULN or total bilirubin > 3–10 × ULN	Creatinine > 3–6 × ULN or >3 × baseline	Symptoms at rest or minimal activity, cardiac biomarkers > ULN
Grade 4	Grade 3 plus fever or peritoneal signs consistent with bowel perforation, or ileus, life-threatening	Life-threatening respiratory compromise; urgent intervention indicated (e.g., intubation and ventilation)	AST/ALT > 20 × ULN or total bilirubin > 10 × ULN	Creatinine > 6 × ULN	Moderate to severe decompensation, worsening signs and symptoms, cardiac biomarkers > 3 × ULN

ADL: activities of daily living; ALT: alanine aminotransferase; AST: aspartate aminotransferase; ECG: echocardiogram; IV: intravenous; ULN: upper limit of normal.
